# 《胸部恶性肿瘤围术期静脉血栓栓塞症预防中国专家共识（2018版）》解读之高危患者筛查篇

**DOI:** 10.3779/j.issn.1009-3419.2019.12.02

**Published:** 2019-12-20

**Authors:** 莉红 李, 辉 李

**Affiliations:** 北京，首都医科大学附属北京朝阳医院胸外科 Department of Thoracic Surgery, Beijing Chaoyang Hospital, Capital Medical University, Beijing 100020, China

**Keywords:** 静脉血栓栓塞症, 胸部恶性肿瘤, Caprini风险评估量表, Rogers评估量表, Venous thromboembolism, Thoracic cancer, Caprini risk assessment model, Rogers risk assessment model

## Abstract

静脉血栓栓塞症（venous thromboembolism, VTE）是胸部恶性肿瘤患者围术期较常见的一种并发症，一旦发生，不但影响患者预后，还将占用大量医疗资源，这正逐步引起我们的广泛重视。然而，我国胸外科对于VTE的认识起步较晚，认知度和重视度亦不够，且目前尚缺少针对围术期VTE预防的指南支持。基于目前我国胸外科对于VTE的认识及预防措施现状，中国胸外科静脉血栓栓塞研究协作组发布了国际首部《胸部恶性肿瘤围术期静脉血栓栓塞症预防中国专家共识（2018版）》。本文将对其中胸部恶性肿瘤患者围术期VTE的高危患者筛查进行解读，以便于更好地理解共识相关内容。

## Abstract (en)

Venous thromboembolism (VTE) is a common perioperative complication in patients with thoracic malignant tumor. Once it occurs, it will not only affect the prognosis of patients, but also occupy a lot of medical resources, which is gradually causing our widespread attention. However, the understanding of VTE in thoracic surgery in our country is relatively late, and the recognition and attention are not enough, and there is still a lack of guidance support for perioperative VTE. Based on the current understanding and preventive measures of VTE in thoracic surgery in China, The China National Research Collaborative Group released the first edition of Chinese experts consensus on the perioperative VTE prophylaxis in 2018. This article will interpret the high-risk patients with perioperative VTE in patients with thoracic malignant tumors, in order to provide a better understanding of Chinese experts consensus for readers.

静脉血栓栓塞症（venous thromboembolism, VTE）在临床上主要包括深静脉血栓（deep venous thrombosis, DVT）及肺栓塞（pulmonary embolism, PE）。DVT和PE是指同一疾病在不同发病部位、不同阶段的不同表现，VTE是外科术后的常见并发症及医院内非预期死亡的重要危险因素之一，是恶性肿瘤患者的第二大死亡原因，同时又是一个可以被预防的疾病。据文献^[1, 2]^统计报道，首次住院的恶性肿瘤患者中根据肿瘤部位的不同，VTE的发病率约为3%-12%。2015年美国外科医师学会主持了一项针对5种高发恶性肿瘤手术患者术后并发症的大型回顾性研究，总计74, 361例患者接受了评估，其中包括肺癌患者6, 849例和食管癌患者3, 126例。研究^[3]^发现，肺癌术后VTE发生率为1.8%，食管癌术后则为5.9%，食管癌术后VTE发生率在所统计的5种癌症中最高。我国针对胸外科肺手术术后VTE的发病率亦进行了一项单中心、前瞻性的队列研究^[4]^，结果显示术后VTE的总发生率为11.5%，肺良性疾病患者VTE的发生率为7.0%，肺恶性疾病患者VTE的发生率为15.0%。目前研究^[5, 6]^已经证实肺癌和食管癌均是VTE的高危因素，特别是围术期VTE发生率较高。因此，对胸部恶性肿瘤手术患者进行VTE风险事件的评估，尽早锁定VTE高危人群，对于优化血栓预防的风险-受益比率至关重要。以上这些问题在《胸部恶性肿瘤围术期静脉血栓栓塞症预防中国专家共识（2018版）》（以下简称《共识》）中均进行了简要和明确的说明，并给出了指导性的意见^[7]^。但由于篇幅限制，共识中没有给予详细的解释。本文将针对胸部恶性肿瘤患者围术期VTE高危人群如何进行筛查做全面的解读。

现有的有关VTE风险评估模型有多种，包括Caprini风险评估量表（Caprini risk assessment model, Caprini RAM）、Rogers评分量表、Padua评分量表、4-Element RAM和Khorana评分量表等。所有这些都是专门为评估VTE的风险而设计的评估工具。美国胸科医师学会（American College of Chest Physicians, ACCP）非骨科外科患者VTE预防临床实践指南第9版（ACCP 9）推荐在外科患者中使用Caprini和Rogers评估量表^[8]^。国内也有研究^[9]^表明Caprini和Rogers风险评估模型联合使用可以提高胸外科手术后患者筛选VTE的准确性。因此本文将重点围绕这两种评分标准进行分析。

## 1 Caprini风险评估量表

Joseph A. Caprini是美国的一位外科医生，于1965年毕业于美国德雷塞尔大学（Drexel University）医学院，现任美国埃文斯顿医院和美国西北大学范伯格医学院的教授，迄今共发表VTE相关研究文献约385篇。基于本身的临床经验和已发表的研究结果，Caprini等自20世纪80年代后期开始研究设计了一个极为细致的个体化VTE风险评估量表，即Caprini血栓风险评估量表（模型），用于内科和外科住院患者VTE的风险评估。该风险评估量表于2005年发表，由密西根大学健康系统推荐采用Caprini评分量表作为内外科患者的风险评估模型^[10]^。在2009年又发表了修改版本，改良的Caprini量表不仅可以根据评分很好地进行VTE危险程度分级，还根据隶属不同风险等级推荐了相应的预防措施，具体包括预防措施的类型及持续时间等，这对于临床医生来说既方便又实用^[11]^。目前基于该量表，已开发出一种新型的电子预警工具——VTE风险计算器，通过根据患者自身状况去选择患者相关风险因素和与手术相关风险因素，可直接计算并显示患者风险等级，并根据患者危险程度推荐相应的预防方法。在西方发达国家，该评估量表已经被很多个人和组织采用，并被翻译成12种语言发表^[12]^。

Caprini RAM目前是外科临床最常用的RAM，是个体化的VTE风险评估量表，目前应用范围很广，包括普通外科、骨科、妇产科以及肿瘤科等。为验证该量表的适用性及有效性，国内外针对此做了大量实验研究。美国密歇根大学卫生系统的研究者利用Caprini RAM对8, 216例普通外科、血管外科和泌尿外科住院患者进行回顾性分析并进行VTE危险程度的分级，结果显示：在手术后30 d内，住院患者总VTE发生率为1.4%，各个危险等级VTE发生率分别为极高危组1.94%、高危组0.97%、中危组0.70%、低危组0.00%，验证了分级程度越高、VTE评分越高，VTE的发生率相对也越高，这说明该量表在这一研究人群中预测VTE的发生是有效的^[11]^。波士顿大学的研究者利用Caprini RAM对2005年-2013年接受肺癌切除术的232例胸外科患者进行了一项回顾性分析，以确定VTE事件发生的频率并评估该模型是否可以对胸外科患者进行危险分层。结果表明：术后60 d VTE发生率为5.2%（12/232），其中低危、中危、高危的VTE发生率分别为0.0%、1.7%和10.3%，VTE发生率随着Caprini评分的升高而升高，这也验证了Caprini RAM在胸外科患者中预测VTE发生率是可行的^[13]^。随后，该大学针对所有胸外科手术患者又进行了一项关于实施Caprini RAM进行VTE风险评估及指导预防方案的前瞻性研究。该研究使用Caprini RAM对126例胸部外科手术患者进行危险程度分级，根据患者不同VTE危险分级对患者使用不同的预防措施，结果提示：有效病例125例，其中高危组24例（19.2%），中危组60例（48.0%），低危组41例（32.8%）。按照RAM方案，高危患者术后30 d每日应用依诺肝素预防，中危患者术后10 d每日应用依诺肝素预防，术后总VTE发生率为2.3%，这提示实施VTE风险评估方案，延长高危患者的预防疗程，对于胸外科患者来说是安全且可行的^[14]^。国内亦有研究^[15]^证明Caprini模型可用于国内综合医院血栓高危住院患者的VTE的风险评估。首都医科大学附属北京朝阳医院胸外科采用Caprini RAM对胸外科肺手术术后VTE的发病率进行了一项研究，结果显示术后VTE的总发生率为11.5%，并且低、中、高危组术后VTE发生率分别为0.0%、12.3%、40.0%^[4]^。随后，该院针对肺癌手术术后VTE相关危险因素进行了一项进一步的单中心研究，该研究针对339例接受肺癌手术的患者进行相关性危险因素回归分析，结果提示第1秒用力呼气容积（forced expiratory volume in one second, FEV_1_）、手术方式以及下肢肌间静脉扩张是肺癌术后合并VTE的独立危险因素^[21]^。这与Caprini RAM模型结论相一致。综上所述，对于国内外胸外科住院患者均可使用Caprini RAM进行VTE危险分层，锁定高危人群，并根据患者不同VTE危险分级对患者使用不同的预防措施。

经典的Caprini RAM包括大约40个危险因素（包括先天性和/或后天性危险因素），几乎涵盖了住院患者VTE的所有危险因素，每个危险因素根据危险程度的不同被赋予1分-5分不同的分值，最后根据得到的累计分数将患者VTE发生风险分为极低危（0分）、低危（1分-2分）、中危（3分-4分）和高危（≥5分）4个等级，不同的风险等级推荐不同的VTE预防措施。因此，该量表不仅可以预测VTE的发生风险，而且还推荐相应的预防措施，包括特定预防措施的类型和持续时间。但在临床实践中，这种经典Caprini RAM标准并不适合胸部恶性肿瘤患者，因为按此标准，几乎所有住院的胸部恶性肿瘤患者都达到了高危风险。因此近年来，一种改良的Caprini RAM已经被国外胸外科医师所使用（表 1）^[16]^。改良的Caprini RAM将风险分级简化为3个级别：低危（0分-4分）、中危（5分-8分）和高危（≥9分），被认为更适用于胸部恶性肿瘤患者，这在《共识》中亦有具体介绍。有学者使用改良的Caprini风险评估模型对接受肺癌手术的患者进行VTE的风险评估，结果显示，当以分值≥9分为高危组的分界线时，其预测的特异度、灵敏度及准确性分别为60.5%、83.3%及61.6%^[13]^。

**1:** 改良Caprini量表（VTE风险评估） Modified Caprini risk assessement model

Caprini risk factor	Caprini score
Age 40 yr-59 yr	1
Abnormal pulmonary function (COPD)	1
Acute myocardial infarction < 1 mo	1
Body mass index ≥30 kg/m^2^	1
Congestive heart failure < 1 mo	1
History of inflammatory bowel disease	1
History of prior major surgery < 1 mo	1
Complications of pregnancy	1
Oral contraceptive use or HRT	1
Sepsis < 1 mo	1
Serious acute lung disease < 1 mo	1
Swollen legs (current)	1
Varicose veins	1
Age 60 yr-74 yr	2
Central venous access	2
Confined to bed > 72 h	2
Major open surgery ≥45 min	2
Present cancer	2
Prior cancer, except nonmelanoma skin	2
Age ≥75 yr	3
History of VTE	3
Family history of VTE	3
Chemotherapy	3
Positive anticardiolipin antibody	3
Positive Lupus anticoagulant	3
Acute spinal cord injury < 1 mo	5
Major surgery ≥6 h	5
Total scores	
Low risk: 0-4 scores; Moderate risk: 5-8 scores; High risk: ≥9 scores; VTE: venous thromboembolism.	

## 2 Rogers评估量表

该量表由来自于Brigham and Women' s Hospital（波士顿）的Rogers于2007年建立（表 2），并发表于《美国外科医师学会杂志》，建立该模型的数据来源于2002年-2004年来自于128个医疗中心的183, 069例外科患者。该研究通过对183, 069例血管外科和普通外科手术的患者使用多元Logistic回归分析方法确定患者术后VTE的高危独立因素，建立一个模型，以识别出术后VTE的高危患者，并确定适当的围术期预防措施^[17]^。但是该模型缺少充分的临床研究验证其有效性及适用性。美国胸外科医师协会在2012年发表的第9版《非骨科手术抗栓及溶栓治疗循证医学临床实践指南》中采纳了Caprini和Rogers风险评估模型，但是该指南评价Rogers模型不够简单易用，该模型本身也具有局限性，其纳入分析的患者并不全是未采取预防措施的患者，其包含部分已采取VTE预防措施的患者。但是我国亦有研究^[9]^证明Caprini和Rogers风险评估模型联合使用在胸外科手术后患者中筛选VTE是有效的，并能提高其准确性。但是单独使用Rogers模型可能不太适用于胸外科手术患者，而通过联合使用两种风险评估模型，发现其准确性均高于单独使用任一模型的准确性。

**2:** Rogers评估量表（VTE风险评估） Rogers risk assessement model

Risk factor	Risk score
Operation type other than endocrine	
Respiratory and hemic	9
Thoracoabdominal aneurysm, embolectomy/ thrombectomy, venous reconstruction, and endovascular repair	7
Aneurysm	4
Mouth, palate	4
Stomach, intestines	4
Integument	3
Hernia	2
ASA physical status classification	
3, 4 or 5	2
2	1
1	0
Work RVU	
> 17	3
10-17	2
< 10	0
Female gender	1
Male gender	0
Disseminated cancer	2
Chemotherapy for malignancy within 30 d of operation	2
Preoperative serum sodium (145 mmol/L)	2
Transfusion (4 U packed RBCs in 72 h before operation)	2
Ventilator-dependent	2
Wound class (clean/contaminated)	1
Preoperative hematocrit (38%)	1
Preoperative bilirubin (> 1.0 mg/dL)	1
Dyspnea	1
Albumin (3.5 mg/dL)	1
Emergency	1
Total scores	
ASA: American Society of Anesthesiologists; RBC: red blood cell; RVU: relative value unit. Low risk: < lt; 7; Moderate risk: 7-10; High risk: > 10.	

VTE是一种常见病，据统计，普通人群中发病率为1/1, 000-3/1, 000^[18]^，在没有任何预防措施的前提下，内科及外科住院患者中VTE的发病率更是高达10%-40%，全球每年确诊的PE和DVT患者约数百万人，目前这已成为世界性的公共健康医疗保健问题。其中，在西方国家PE被称之为常见的三大心血管疾病之一，因“发病率高、死亡率高、漏诊率高”，也被称作“21世纪亟待解决的十大心肺疾病之一”。VTE一旦发生，不但影响患者的预后，存在致死及致残的风险，还将占用大量医疗资源，严重影响患者的生活质量。尽管如此，它仍是“最有可能预防的一种致死性疾病”。我国对于VTE的认识起步较晚，目前尚且缺少VTE相关的流行病学资料，且仍有部分胸外科医师对术后VTE事件的认知度和重视度不够^[19]^。基于目前我国胸外科对于VTE的认识及预防措施现状，应推荐常规使用相关量表，加强针对围术期患者高危因素的筛查，以尽早锁定VTE高危患者，早期采取适当的血栓预防措施，指导后续临床治疗，改善患者预后和生活质量，减少医疗费用。

然而目前虽然有大量的国外研究及初步实践证明Caprini血栓风险评估量表是一个安全有效、简单可行和经济实用的VTE风险预测工具，但是其主要适用于西方发达国家，究其是否适用于我国胸外科患者尚有待进一步证实及商榷。这就需要我们根据中国的国情及中国患者的临床特点研发出一种更为简洁实用而又安全有效的评分标准。目前我们已进行了相关研究并尝试建立了一种VTE风险评估模型，并验证该模型在评估患者VTE事件风险方面是有效的，且其在一定程度上优于Caprini RAM^[20]^。然而该模型只在一个大型研究中心被验证，这限制了它的有用性及被广泛使用性，我们希望它在给中国患者带来益处的同时，该模型能在一个大型多中心的回顾性的研究中得到进一步的验证和修正。
